# Monocyte IL-1β predicts adverse cardiovascular events and associates with coronary microvascular dysfunction in kidney transplant recipients

**DOI:** 10.3389/fcvm.2025.1689566

**Published:** 2026-01-07

**Authors:** Zhengwei Yin, Qingyan Yang, Jianle Han, Junwei Yang, Tao Li, Jingjun Suo, Shuaiping Yang, Xiaobo Wang, Shuailin Zhao, Chang’an Wang

**Affiliations:** Kidney Transplantation and Nephrology Treatment Center, The Seventh People’s Hospital of Zhengzhou, Zhengzhou, China

**Keywords:** coronary microvascular dysfunction, interleukin 1β, kidney transplant recipient, prognosis, pyroptosis

## Abstract

**Aims:**

Cardiovascular disease remains the leading cause of death after kidney transplantation. Coronary microvascular dysfunction (CMD) is common in kidney transplant recipients (KTRs), prognostically informative for cardiovascular events, and tightly related to inflammation. We aimed to test whether pretransplant monocytic expression of pyroptosis-related genes (*IL-1β*, *GSDMD*, *Caspase-1*, and *NLRP3*) independently predicts long-term mortality and major adverse cardiovascular events (MACE) in KTRs, and to evaluate its associations with CMD.

**Methods:**

We enrolled 305 KTRs. Monocytes were isolated preoperatively and qPCR quantified the four genes (normalized to GAPDH). MACE included death, myocardial infarction, stroke, and heart failure. Multivariable Cox regression was used to adjust for confounders associated with prognosis. CMD was evaluated using coronary flow reserve (CFR) in 41 KTRs and serum syndecan-1 levels (an endothelial injury marker) in 88 KTRs.

**Results:**

Over a median 4.0 years, 62/305 (20.3%) had MACE. *IL-1β* expression was higher in those with events. In Cox models with *IL-1β* entered as a standardized continuous variable (per SD), *IL-1β* independently predicted death (adjusted HR 1.530, 95%CI 1.165–2.009) and MACE (adjusted HR 1.622, 95%CI 1.283–2.052). When modeled categorically as tertiles, the highest vs. lowest *IL-1β* tertile conferred greater risk (death, adjusted HR 3.771, 95%CI 1.516–9.384; MACE, adjusted HR 4.398, 95%CI 2.003–9.654). *IL-1β* correlated inversely with CFR (R = −0.40, *P* = 0.009) and positively with syndecan-1 (R = 0.47, *P* < 0.001). Other genes showed weaker or nonsignificant associations.

**Conclusion:**

*IL-1β* is a robust, independent predictor of death and MACE in KTRs. Its associations with impaired CFR and elevated syndecan-1 support a mechanistic link to CMD.

## 1 Introduction

Kidney transplantation (KT) is the optimal treatment for patients with end-stage kidney disease (ESKD), significantly improving both survival and quality of life (1, 2). Despite this, cardiovascular disease remains the leading cause of mortality in kidney transplant recipients (KTRs), with rates of major adverse cardiovascular events (MACE) far exceeding those in the general population (1–7). The elevated cardiovascular risk in KTRs is not fully explained by traditional risk factors alone, suggesting the involvement of other pathological mechanisms unique to the uremic and post-transplant environment (1).

Growing evidence implicates coronary microvascular dysfunction (CMD) as a key contributor to adverse cardiovascular outcomes (8–15). CMD, characterized by impaired coronary blood flow regulation in the absence of obstructive epicardial coronary artery disease (CAD) (8, 10), is highly prevalent among patients with chronic kidney disease and KTRs (16–20). Therefore, CMD may represent a crucial pathophysiological link to the heightened incidence of MACE observed after kidney transplantation.

Inflammation is a kye pathogenic driver of CMD (21–26). Pyroptosis, a highly pro-inflammatory form of regulated cell death, has recently emerged as a critical mechanism in the pathogenesis of various cardiovascular diseases (27, 28). This process is typically initiated by the activation of an inflammasome, such as the *NLRP3* inflammasome, which leads to the activation of *Caspase-1* (29–31). Active *Caspase-1* then cleaves gasdermin D (*GSDMD*) to form pores in the cell membrane and processes pro-interleukin-1β (pro-*IL-1β*) into its mature, secreted form, thereby amplifying the inflammatory cascade (27–31). Monocytes and macrophages, key players in vascular inflammation, are primary effectors of the pyroptotic pathway (29).

However, it remains unknown whether the pyroptotic activity in peripheral blood monocytes prior to surgery can serve as a biomarker for future cardiovascular risk in KTRs. Therefore, the primary aim of this study was to investigate whether the preoperative expression levels of pyroptosis-related genes (*IL-1β*, *GSDMD*, *Caspase-1*, and *NLRP3*) in circulating monocytes are independent predictors of long-term MACE in KTRs. A secondary objective was to explore the association between the expression of these genes and the status of coronary microvascular function.

## 2 Materials and methods

### 2.1 Human subjects and sample collection

In this study, we recruited 305 KTRs between January 2018 and December 2022 at the Department of Kidney Transplantation and Nephrology Treatment Center, The Seventh People's Hospital of Zhengzhou. The exclusion criteria were: (1) severe liver impairment; (2) presence of severe, active infections; and (3) a history of MACE within the 2 weeks prior to hospital admission. All transplant recipients were consecutively recruited before the kidney transplant surgery, and all had already initiated maintenance dialysis at the time of enrollment. We also enrolled 87 healthy individuals without chronic kidney disease or known cardiovascular disease, who were undergoing routine health check-ups, to serve as a control group.

Peripheral blood samples were collected from all KTRs before the kidney transplant surgery. Monocytes were enriched from whole blood using the RosetteSep™ Human Monocyte Enrichment Cocktail (STEMCELL Technologies, Vancouver, Canada) according to the manufacturer's protocol. Serum was concurrently collected from peripheral blood by centrifugation and stored at −80°C until use.

Clinical data were collected preoperatively and included demographic characteristics, medical history, physical examination findings, and laboratory parameters.

The study protocol was approved by the ethical committee of The Seventh People's Hospital of Zhengzhou. All procedures were conducted in strict accordance with the latest version of the Declaration of Helsinki. Written informed consent was obtained from all participants prior to their inclusion in the study.

### 2.2 RNA isolation and real-time PCR

In this study, the expression levels of four pyroptosis-related genes (*IL-1β*, *GSDMD*, *Caspase-1*, and *NLRP3*) were evaluated using real-time PCR. Total RNA was extracted from enriched monocytes using Trizol® reagent (Invitrogen, Carlsbad, CA) following the manufacturer's instructions. RNA concentration and purity were assessed by measuring absorbance at 260 nm and 280 nm with a Nanodrop ND-3000 spectrophotometer (Thermo Fisher Scientific, Waltham, MA). For each sample, 300 ng of total RNA was reverse transcribed into cDNA using a cDNA synthesis kit (Thermo Fisher Scientific). Real-time quantitative PCR (qPCR) was performed with TaqMan Gene Expression Assays (Applied Biosystems, Foster City, CA) on a StepOnePlus qPCR system (Applied Biosystems).

The following TaqMan probes were used: *IL-1β* (Hs01555410_m1), *GSDMD* (Hs00986748_g1), *Caspase-1* (Hs00354836_m1), and *NLRP3* (Hs00918082_m1). The expression of the four target genes was normalized to the housekeeping gene GAPDH (Hs02758991_g1). The relative expression of each gene was calculated using the 2-ΔΔCt method. Expression levels in KTRs were presented as a fold change relative to the mean expression level in the 87 healthy controls.

### 2.3 Endpoints

The primary endpoint was MACE, defined as a composite of all-cause mortality, nonfatal myocardial infarction, nonfatal stroke, or hospitalization due to heart failure (HF). Detailed definitions for each component of the endpoint are provided in Supplementary Table S1. After hospital discharge, patients were followed up at 1 month, 6 months, 1 year, and annually thereafter for up to 6 years, through clinic visits or telephone interviews.

### 2.4 Enzyme-linked immunosorbent assay (ELISA) of syndecan-1

The previous study have shown that circulating syndecan-1 levels reflect endothelial glycocalyx shedding and are associated with CMD (32). To assess whether monocytic gene expression relates to CMD, we measured serum syndecan-1 concentrations in 88 KTRs (enrolled from June 2021 to December 2022) using a commercial ELISA kit (Human Syndecan-1 ELISA Kit, Abcam, Cambridge, UK) according to the manufacturer's protocol.

### 2.5 Coronary flow reserve (CFR) measurement

To directly assess coronary microvascular function, we consecutively recruited KTRs for CFR assessment from August 2021 onwards. The main reasons for not completing analyzable CFR in some patients were (1) refusal to undergo the additional imaging and (2) technically inadequate Doppler signals despite acquisition attempts. After excluding examinations with inadequate image quality, a total of 41 KTRs remained for the CFR analysis.

As prevous reported (17, 18), following a baseline echocardiographic evaluation, dipyridamole was infused at a rate of 0.84 mg/kg over 6 min. CFR was calculated as the ratio of hyperemic to basal peak diastolic coronary flow velocity in the left anterior descending artery. Patients were instructed to abstain from methylxanthine-containing beverages for at least 24 h prior to the study.

### 2.6 Statistical analysis

Continuous variables were presented as mean ± standard deviation (SD) for normally distributed data or median with interquartile range (IQR) for non-normally distributed data. Comparisons between two groups were performed using the independent sample *t*-test or Mann–Whitney *U*-test. Categorical variables were expressed as frequencies (percentages) and compared using the chi-square test or Fisher's exact test.

For Kaplan–Meier survival analysis, KTRs were categorized into tertiles based on the expression level of each gene. Kaplan–Meier curves were generated to visualize time-to-first-event data, and differences were assessed with the log-rank test.

Univariate and multivariable Cox proportional-hazards regression models were used to calculate hazard ratios (HRs) and 95% confidence intervals (CIs). The multivariable models were adjusted for potential confounders associated with prognosis, including age, sex, diabetes, hypertension, first kidney transplant, living donor kidney transplantation (LDKT), causes of ESKD (primary glomerulonephritis vs. others), cytomegalovirus serostatus [Donor (D) and Recipient (R): D+/R- D+/R+ vs. D-/R- D-/R+], proteinuria (trace/+/++ vs. +++/++++), dialysis vintage, and history of CAD or HF. Restricted cubic splines (RCS) regression models with four knots (two internal nodes) at the 0th, 35th, 65th, and 100th percentiles were used to assess potential nonlinear associations.

A baseline prognostic model was constructed using the aforementioned confounders. The incremental predictive value of adding *IL-1β* expression to the baseline model was assessed by comparing Harrell's concordance index (C-index) and calculating the change in C-index (ΔC-index) with 2000 bootstrap replications.

Spearman's correlation was used to analyze relationships between variables.

Multiple comparisons across the four pyroptosis-related genes were adjusted for using the Bonferroni method.

A two-sided *P*-value <0.05 was considered statistically significant. All analyses were performed using SPSS (Version 22.0, IBM Corp.) and R (Version 4.2.0, R Foundation for Statistical Computing).

## 3 Results

### 3.1 Baseline characteristics

The baseline clinical and demographic characteristics of the study population are presented in Table 1. Of the 305 KTRs, 62 (20.3%) experienced MACE during a median follow-up of 4 years (interquartile range: 2.4–5.0 years). Compared to KTRs without MACE, those who developed MACE were significantly older, had a higher prevalence of diabetes, and exhibited lower baseline eGFR (all *P* < 0.05). All other baseline characteristics were comparable between groups—including sex, hypertension, smoking, transplant-related factors, ESKD etiology, induction regimen, CMV serostatus, proteinuria, and prior CAD/HF (all *P* > 0.05).

**Table 1:** Baseline characteristics of the study population.

Variables	Healthy controls (*n* = 87)	Total KTR (*n* = 305)	*P*-value	KTR without MACE (*n* = 243)	KTR with MACE (*n* = 62)	*P*-value
Age, years	46.7 ± 11.5	50.9 ± 11.7	0.003	49.84 (11.70)	54.87 (10.95)	0.002
Female, *n* (%)	30 (34.4)	117 (38.4)	0.51	98 (40.3)	19 (30.6)	0.21
Diabetes, *n* (%)	11 (12.6)	70 (23.0)	0.036	49 (20.2)	21 (33.9)	0.034
Hypertension, *n* (%)	16 (18.3)	252 (82.6)	<0.001	196 (80.7)	56 (90.3)	0.109
Current smoking, *n* (%)	23 (26.4)	94 (30.8)	0.43	79 (32.5)	15 (24.2)	0.266
First transplant, *n* (%)	–	272 (89.2)	–	219 (90.1)	53 (85.5)	0.412
LDKT, *n* (%)	–	40 (13.1)	–	33 (13.6)	7 (11.3)	0.79
Causes of ESKD, *n* (%)						
Primary glomerulonephritis		234 (76.7)		183 (75.3)	51 (82.3)	0.592
Diabetic nephropathy		21 (6.9)		16 (6.6)	5 (8.1)	
Polycystic kidney disease		11 (3.6)		10 (4.1)	1 (1.6)	
Lupus nephritis		9 (3.0)		8 (3.3)	1 (1.6)	
Allergic purpura nephritis		7 (2.3)		7 (2.9)	0 (0.0)	
Others		23 (7.5)		19 (7.8)	4 (6.5)	
Induction agent, *n* (%)						
Thymoglobulin		211 (69.2)		169 (69.5)	42 (67.7)	0.963
Basiliximab		80 (26.2)		63 (25.9)	17 (27.4)	
Others		14 (4.6)		11 (4.5)	3 (4.8)	
Cytomegalovirus serostatus, *n* (%)						
D+/R-		27 (8.9)		22 (9.1)	5 (8.1)	0.155
D+/R+		187 (61.3)		145 (59.7)	42 (67.7)	
D-/R+		81 (26.6)		70 (28.8)	11 (17.7)	
D-/R-		10 (3.3)		6 (2.5)	4 (6.5)	
Proteinuria, *n* (%)						
Trace		31 (10.2)		25 (10.3)	6 (9.7)	0.253
1+		87 (28.5)		65 (26.7)	22 (35.5)	
2+		110 (36.1)		95 (39.1)	15 (24.2)	
3+		55 (18.0)		41 (16.9)	14 (22.6)	
4+		22 (7.2)		17 (7.0)	5 (8.1)	
Dialysis vintage	–	23.3 ± 10.2	–	23.2 ± 10.6	23.9 ± 8.5	0.619
Previous CAD, *n* (%)	–	59 (19.3)	–	45 (18.5)	14 (22.6)	0.587
Previous HF, *n* (%)	–	48 (15.7)	–	35 (14.4)	13 (21.0)	0.284
IL-1b	0.91 (0.69–1.19)	1.29 (1.08–1.55)	<0.001	1.25 (1.04–1.50)	1.48 (1.27–1.68)	<0.001
GSDMD	0.92 (0.66–1.27)	1.50 (1.16–1.98)	<0.001	1.46 (1.13–1.93)	1.74 (1.33–2.24)	0.028
Caspase-1	0.94 (0.70–1.21)	1.55 (1.16–2.09)	<0.001	1.54 (1.17–2.02)	1.62 (1.13–2.20)	0.68
NLRP3	0.87 (0.65–1.28)	1.16 (0.88–1.52)	<0.001	1.16 (0.88–1.52)	1.17 (0.89–1.49)	0.863

### 3.2 Expression of the four pyroptosis-related genes

**Figure 1:**
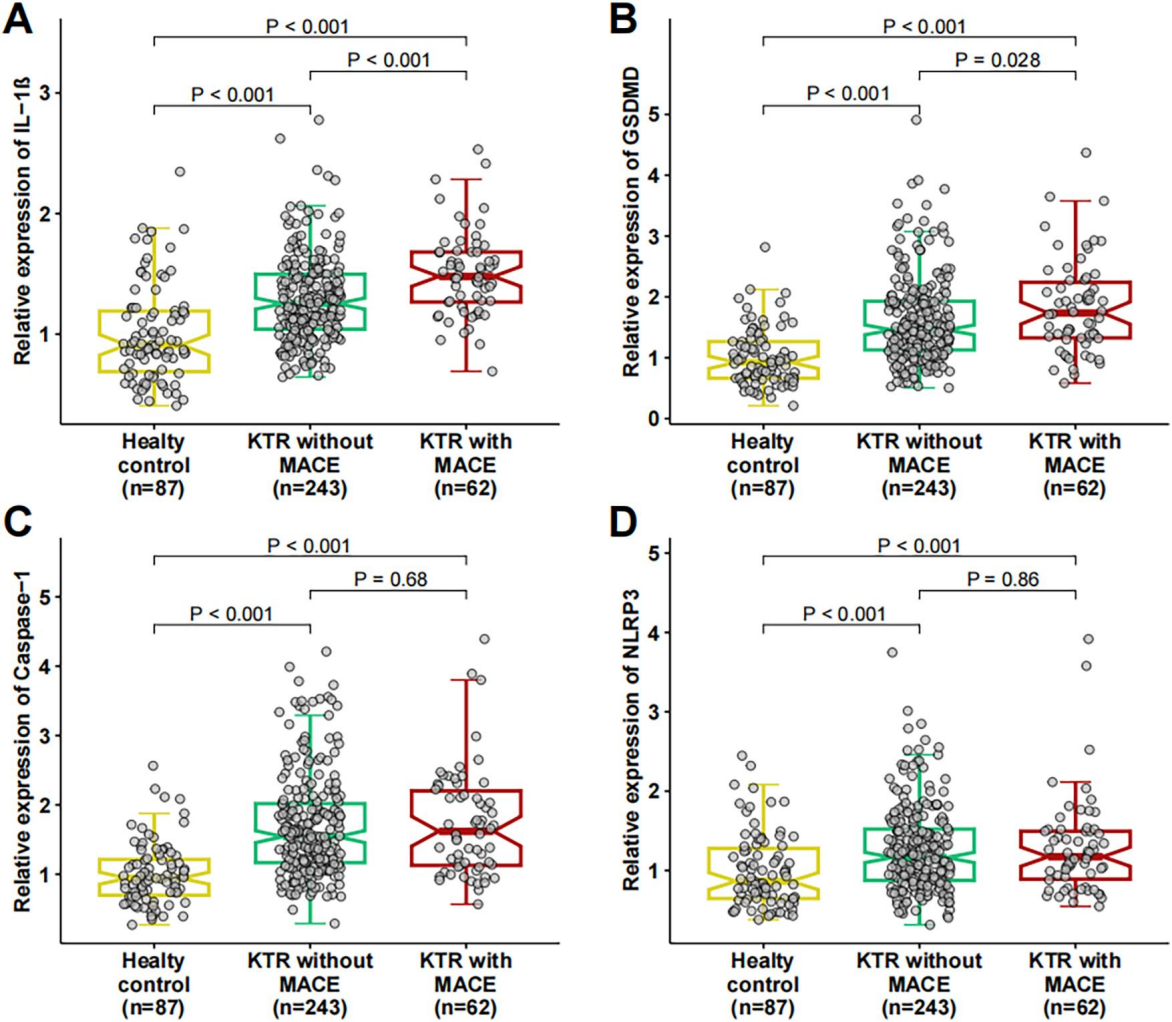
Expression of pyroptosis-related genes in healthy controls and kidney transplant recipients. Boxplots showing the relative mRNA expression of **(A)** *IL-1β*, **(B)** *GSDMD*, **(C)** *Caspase-1*, and **(D)** *NLRP3* in monocytes. Comparisons are made between healthy controls (*n* = 87), kidney transplant recipients (KTR) without major adverse cardiovascular events (MACE) (*n* = 243), and KTR with MACE (*n* = 62). The horizontal line within the box represents the median, the box boundaries represent the interquartile range, and whiskers extend to 1.5 times the interquartile range. *P*-values were calculated using the Mann–Whitney *U*-test.

As shown in Figure 1 and Table 1, the expression levels of all four pyroptosis-related genes (*IL-1β*, *GSDMD*, *Caspase-1*, and *NLRP3*) were significantly elevated in KTRs (with or without MACE) compared to healthy controls (all *P* < 0.001). Among KTRs, the expression of *IL-1β* and *GSDMD* was significantly higher in patients who subsequently developed MACE compared to those who did not [*IL-1β*: 1.48 (1.27–1.68) vs. 1.25 (1.04–1.50), *P* < 0.001; *GSDMD*: 1.74 (1.33–2.24) vs. 1.46 (1.13–1.93), *P* = 0.028]. No significant differences were observed for *Caspase-1* and *NLRP3* expression between the KTR groups with and without MACE (*P* > 0.05).

After applying Bonferroni correction for multiple testing (significance threshold *P* < 0.0125 for the four genes), IL-1*β* expression remained significantly higher in KTRs with MACE than in those without MACE, whereas the difference in GSDMD expression between the two groups was no longer statistically significant.

### 3.3 Kaplan–Meier survival analyzing the prognostic significance of the four pyroptosis-related genes

**Figure 2:**
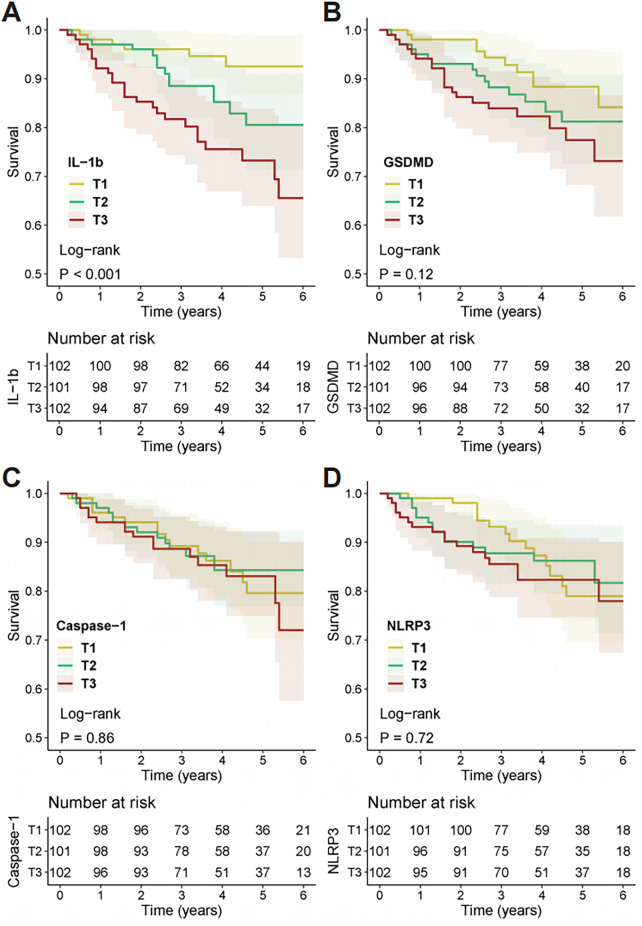
Kaplan–Meier analysis of all-cause mortality stratified by pyroptosis-related gene expression. Survival curves for all-cause mortality are shown for kidney transplant recipients categorized into tertiles (T1, T2, T3) based on the expression of **(A)** *IL-1β*, **(B)** *GSDMD*, **(C)** *Caspase-1*, and **(D)** *NLRP3*. Tertile 1 (T1) represents the lowest expression, and Tertile 3 (T3) represents the highest. *P*-values were determined using the log-rank test. The tables below each plot indicate the number of patients at risk in each tertile at specific time points.

**Figure 3:**
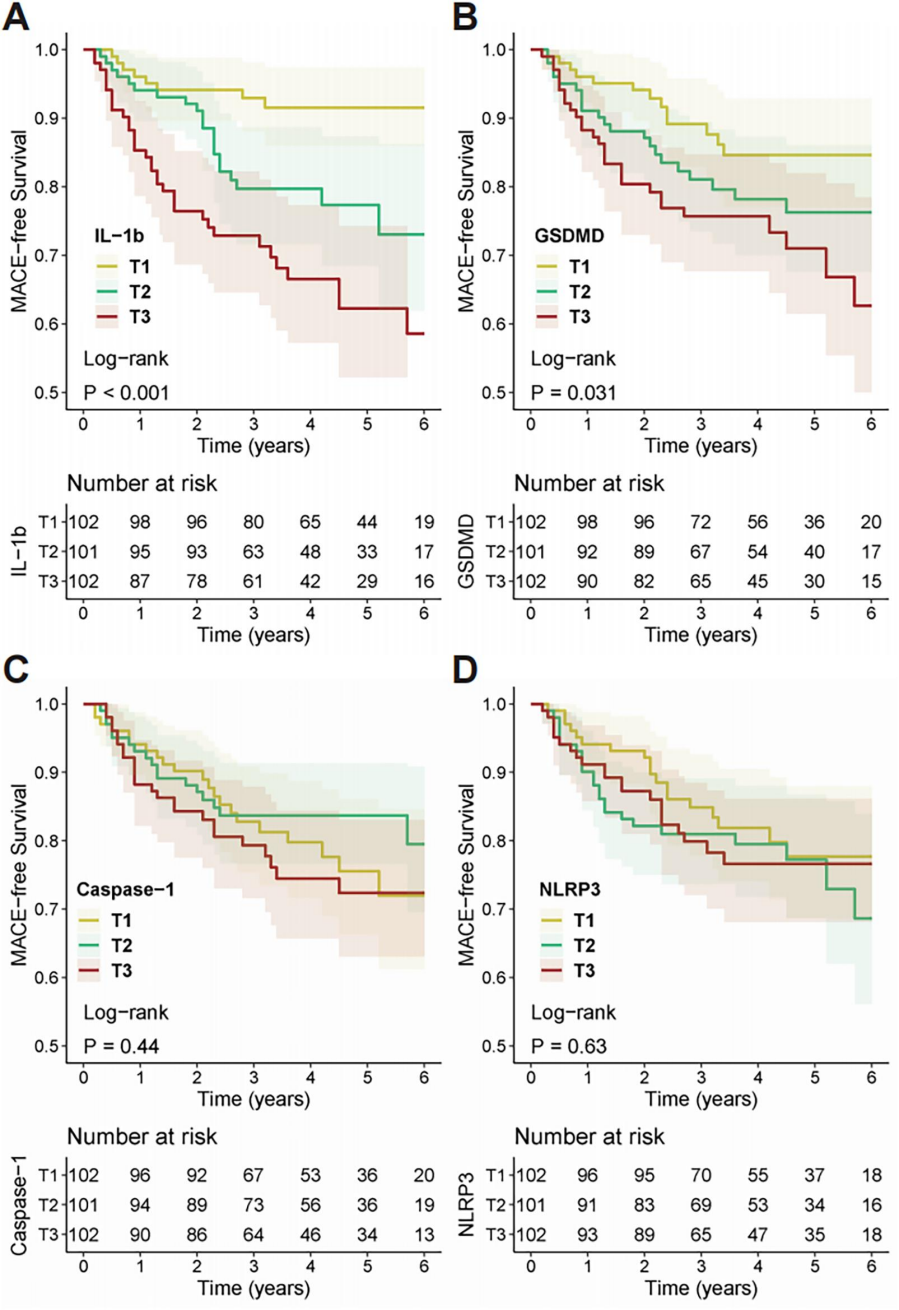
Kaplan–meier analysis of MACE-free survival stratified by pyroptosis-related gene expression. Survival curves for MACE-free survival are shown for kidney transplant recipients categorized into tertiles (T1, T2, T3) based on the expression of **(A)** *IL-1β*, **(B)** *GSDMD*, **(C)** *Caspase-1*, and **(D)** *NLRP3*. Tertile 1 (T1) represents the lowest expression, and Tertile 3 (T3) represents the highest. *P*-values were determined using the log-rank test. The tables below each plot indicate the number of patients at risk in each tertile at specific time points.

Kaplan–Meier survival analysis was performed to assess the prognostic significance of the four genes. Based on tertiles of the relative expression for each gene, patients were divided into three groups—T1 (low), T2 (medium), and T3 (high). Kaplan–Meier survival curves (Figures 2, 3) revealed that KTRs with higher *IL-1β* expression levels had significantly worse clinical outcomes for both all-cause mortality (Log-rank test, *P* < 0.001) and MACE (Log-rank test, *P* < 0.001). In contrast, KTRs with higher *GSDMD* expression levels showed no significant difference in all-cause mortality (Log-rank test, *P* = 0.12), but had worse prognosis for MACE (Log-rank test, *P* = 0.031). Furthermore, there were no statistically significant differences in all-cause mortality or MACE outcomes for KTRs with varying levels of *Caspase-1* (Death: Log-rank test, *P* = 0.86; MACE: Log-rank test, *P* = 0.44) or *NLRP3* (Death: Log-rank test, *P* = 0.72; MACE: Log-rank test, *P* = 0.63) expression.

### 3.4 *IL-1β* as an independent predictor for outcomes in KTRs

Kaplan–Meier survival curves indicated that different expression levels of *IL-1β* could predict the prognosis of KTRs. We further used multivariable Cox regression models to adjust for potential confounders associated with prognosis. *IL-1β* expression was included in the Cox models either as a continuous variable (per standard deviation increase) or as a categorical variable defined by tertiles (T1, T2, T3). The adjusted Model 1 controlled for age and sex, while model 2 was further adjusted for diabetes, hypertension, first kidney transplant, LDKT, causes of ESKD, cytomegalovirus serostatus, proteinuria, eGFR, dialysis vintage, and history of CAD or HF.

When *IL-1β* expression was treated as a continuous variable in the Cox regression model, higher levels of *IL-1β* were significantly associated with both all-cause mortality (Table 2: Per-SD: Unadjusted Model: HR = 1.579, 95% CI = 1.235–2.019, *P* < 0.001; Adjusted Model 1: HR = 1.565, 95% CI = 1.211–2.023, *P* = 0.001; Adjusted Model 2: HR = 1.530, 95% CI = 1.165–2.009, *P* = 0.002) and MACE (Per-SD: Unadjusted Model: HR = 1.602, 95% CI = 1.301–1.973, *P* < 0.001; Adjusted Model 1: HR = 1.597, 95% CI = 1.288–1.980, *P* < 0.001; Adjusted Model 2: HR = 1.622, 95% CI = 1.283–2.052, *P* < 0.001).

**Table 2:** Univariate and multivariable Cox regression analyses of the association between continuous *IL-1β* expression and clinical outcomes.

Events	Continuous	Unadjusted model		Adjusted model 1		Adjusted model 2	
		HR 95% CI	*P*-value	HR 95% CI	*P*-value	HR 95% CI	*P*-value
Death	Per 1-SD	1.579 (1.235–2.019)	<0.001	1.565 (1.211–2.023)	0.001	1.530 (1.165–2.009)	0.002
MACE	Per 1-SD	1.602 (1.301–1.973)	<0.001	1.597 (1.288–1.980)	<0.001	1.622 (1.283–2.052)	<0.001

Adjusted Model 1: adjusting for age and sex.

Adjusted Model 2: adjusting for age, sex, diabetes, hypertension, first kidney transplant, living donor kidney transplantation (LDKT), causes of end-stage kidney disease (ESKD) (primary glomerulonephritis vs. others), cytomegalovirus serostatus [Donor (D) and Recipient (R): D+/R- D+/R+ vs. D-/R- D-/R+], proteinuria (trace/+/++ vs. +++/++++), dialysis vintage, and history of coronary artery disease (CAD) or heart failure (HF).

When *IL-1β* expression was modeled categorically by tertiles in the Cox regression model, KTRs with the highest *IL-1β* expression (T3) compared to those with the lowest expression (T1) had significantly worse outcomes for both all-cause mortality (Table 3: T3 vs. T1: Unadjusted Model: HR = 4.740, 95% CI = 1.943–11.559, *P* = 0.001; Adjusted Model 1: HR = 4.043, 95% CI = 1.656–9.874 *P* = 0.002; Adjusted Model 2: HR = 3.771, 95% CI = 1.516–9.384, *P* = 0.004) and MACE (T3 vs. T1: Unadjusted Model: HR = 5.059, 95% CI = 2.341–10.933, *P* < 0.001; Adjusted Model 1: HR = 4.563, 95% CI = 2.106–9.887, *P* < 0.001; Adjusted Model 2: HR = 4.398, 95% CI = 2.003–9.654, *P* < 0.001).

**Table 3:** Univariate and multivariable Cox regression analysis of the association between IL-1β expression tertiles and clinical outcomes.

Events	Tertiles	Unadjusted model		Adjusted model 1		Adjusted model 2	
		HR 95% CI	*P*-value	HR 95% CI	*P*-value	HR 95% CI	*P*-value
Death	T2 vs. T1	2.537 (0.975–6.604)	0.056	2.239 (0.859–5.835)	0.099	1.979 (0.750–5.224)	0.168
	T3 vs. T1	4.740 (1.943–11.559)	0.001	4.043 (1.656–9.874)	0.002	3.771 (1.516–9.384)	0.004
MACE	T2 vs. T1	2.755 (1.213–6.257)	0.015	2.552 (1.122–5.802)	0.025	2.411 (1.053–5.522)	0.037
	T3 vs. T1	5.059 (2.341–10.933)	<0.001	4.563 (2.106–9.887)	<0.001	4.398 (2.003–9.654)	<0.001

Adjusted Model 1: adjusting for age and sex.

Adjusted Model 2: adjusting for age, sex, diabetes, hypertension, first kidney transplant, living donor kidney transplantation (LDKT), causes of end-stage kidney disease (ESKD) (primary glomerulonephritis vs. others), cytomegalovirus serostatus [Donor (D) and Recipient (R): D+/R- D+/R+ vs. D-/R- D-/R+], proteinuria (trace/+/++ vs. +++/++++), dialysis vintage, and history of coronary artery disease (CAD) or heart failure (HF).

RCS analysis showed a generally linear association between *IL-1β* levels and the risk of death and MACE, with no significant evidence of nonlinearity (*P* for nonlinear = 0.29 and 0.0986, respectively; Supplementary Figure S1).

Furthermore, adding *IL-1β* expression to the baseline model comprising potential confounders significantly improved the predictive accuracy for both death (C-index increased from 0.699 to 0.731, ΔC-index: 0.032, *P* = 0.008) and MACE (C-index increased from 0.674 to 0.716, ΔC-index: 0.042, *P* < 0.001) (Table 4).

**Table 4:** Incremental predictive value of IL-1β for death and MACE.

Events	C-index (baseline model)	C-index (new model)	ΔC-index (95%CI)	*P*-value
Death	0.699 (0.622–0.776)	0.731 (0.655–0.807)	0.032 (0.010–0.055)	0.008
MACE	0.674 (0.610–0.738)	0.716 (0.655–0.777)	0.042 (0.018–0.065)	<0.001

### 3.5 Association of *IL-1β* with coronary microvascular dysfunction

**Figure 4:**
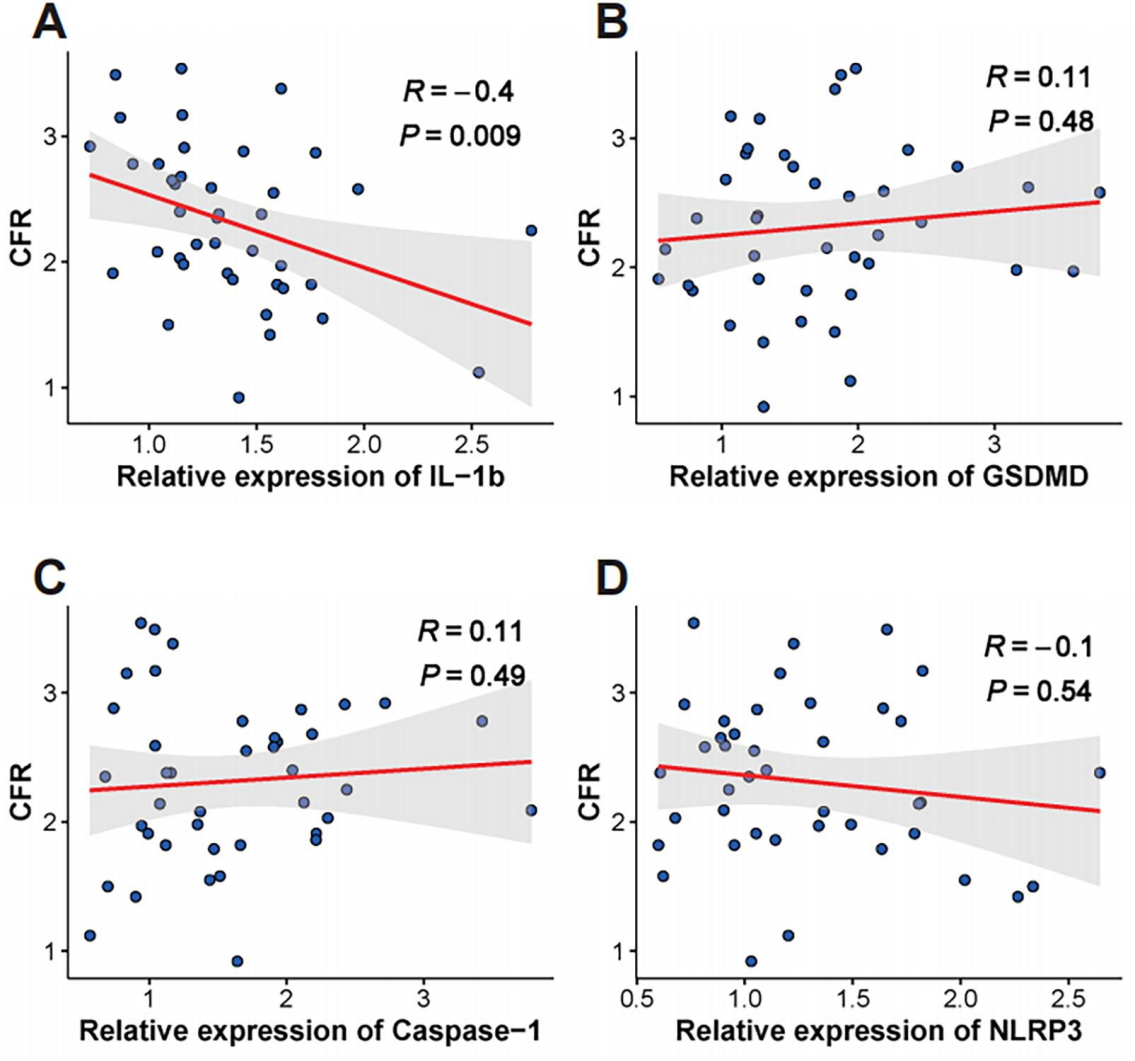
Correlation between pyroptosis gene expression and coronary flow reserve. Scatter plots illustrating the relationship between coronary flow reserve (CFR) and the relative expression of **(A)** *IL-1β*, **(B)** *GSDMD*, **(C)** *Caspase-1*, and **(D)** *NLRP3* in kidney transplant recipients (*n* = 41). The red line represents the linear regression fit, and the gray shaded area indicates the 95% confidence interval. Pearson's correlation coefficient (R) and the corresponding *p*-value are displayed for each analysis.

**Figure 5:**
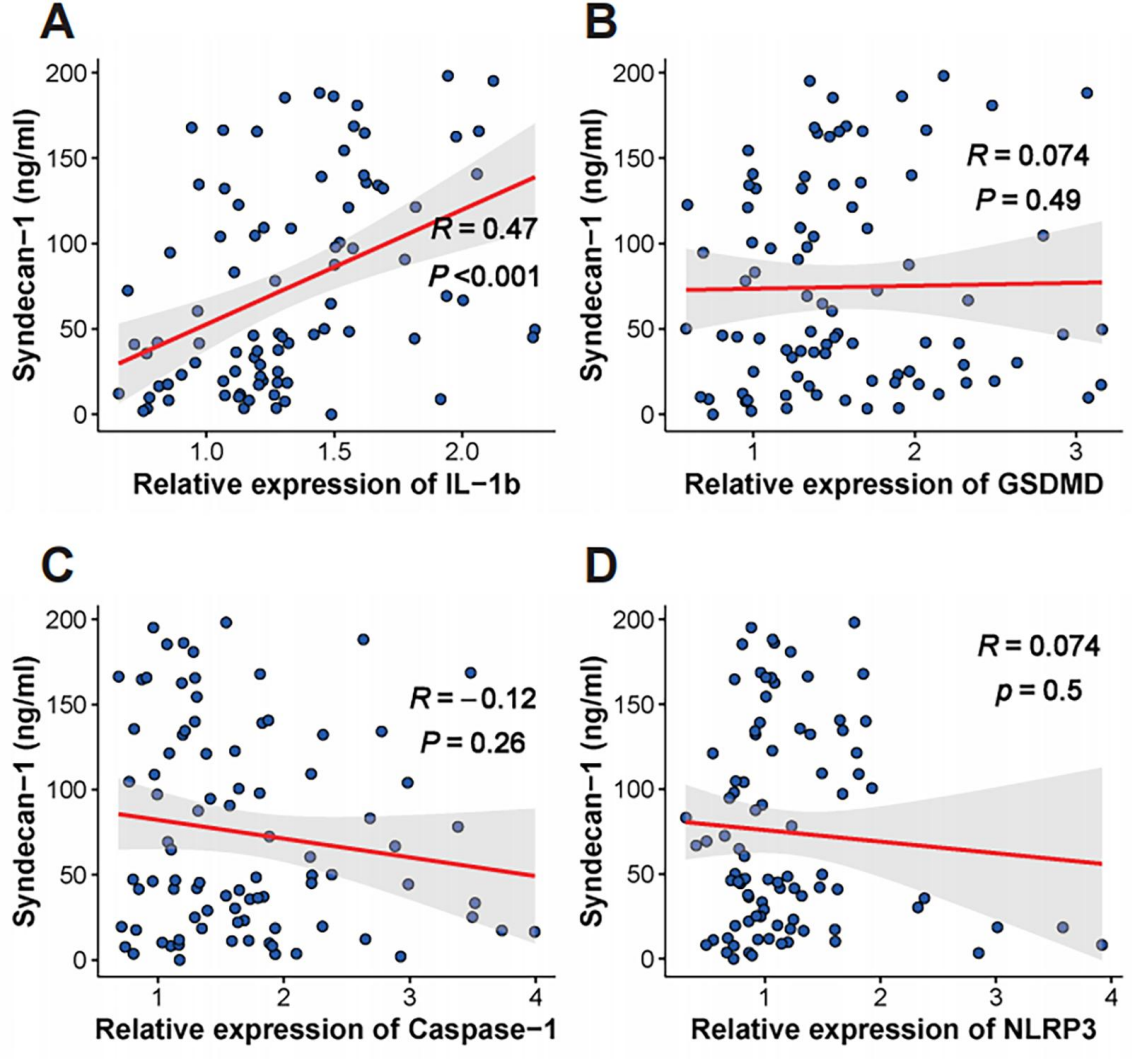
Relationship between pyroptosis gene expression and the endothelial injury marker syndecan-1. Scatter plots show the correlation between serum Syndecan-1 concentration (ng/ml) and the relative mRNA expression of **(A)** *IL-1β*, **(B)** *GSDMD*, **(C)** *Caspase-1*, and **(D)** *NLRP3*. The red line indicates the linear regression line, and the shaded gray area represents the 95% confidence interval. Pearson's correlation coefficient **(R)** and the associated *p*-value are provided for each plot.

Given that CMD markedly increases the risk of MACE in KTRs (16), we hypothesized that higher monocytic *IL-1β* expression contributes to MACE by promoting CMD. Accordingly, we assessed associations between *IL-1β* expression and two indices that reflect CMD severity—CFR and serum syndecan-1. In 41 KTRs, monocytic *IL-1β* expression was the only gene to show a significant negative correlation with CFR (R = −0.4, *P* = 0.009; Figure 4A). In 88 KTRs, *IL-1β* expression demonstrated a strong positive correlation with serum syndecan-1 levels (R = 0.47, *P* < 0.001; Figure 5A). The other pyroptosis-related genes showed no significant correlation with either CFR or syndecan-1.

## 4 Discussion

In this cohort study, we identified that elevated monocytic expression of *IL-1β* is a powerful and independent predictor of both MACE and all-cause mortality. This association remained robust after adjusting for a comprehensive set of traditional cardiovascular and transplant-related risk factors. Furthermore, we demonstrated a significant correlation between *IL-1β* levels, markers of endothelial injury, and impaired coronary microvascular function, providing novel mechanistic insights into the pathophysiology of cardiovascular disease in this vulnerable population.

The principal finding of this study is the clinical utility of *IL-1β* as a prognostic biomarker in KTRs. Cardiovascular disease is the leading cause of death in KTRs (1, 2), yet conventional risk stratification models often fall short (4, 5, 7). Our data show that *IL-1β* provides incremental prognostic value beyond established risk factors, as evidenced by the significant improvement in the C-index for predicting both MACE and death. The continuous and graded relationship between *IL-1β* levels and adverse outcomes, as shown in our Cox and restricted cubic spline analyses, suggests that *IL-1β* could be used to identify high-risk individuals who may benefit from more intensive surveillance, aggressive risk factor management, or targeted anti-inflammatory therapies.

Several potential mechanisms may explain the strong association between elevated *IL-1β* and poor cardiovascular outcomes in KTRs. Our findings suggest a central role for monocyte-mediated pyroptosis, an inflammatory form of programmed cell death (29). We observed significantly higher expression of not only *IL-1β* but also *GSDMD*, the key executioner of pyroptosis (31), in KTRs, particularly in those who developed MACE. This process culminates in the release of potent inflammatory cytokines, including mature *IL-1β*, which drives a systemic inflammatory state (27–31). This inflammation, in turn, likely contributes to cardiovascular pathology through two interconnected pathways. First, we identified a strong positive correlation between *IL-1β* and Syndecan-1, a marker of endothelial glycocalyx shedding and injury. Chronic endothelial damage is a foundational step in the development of atherosclerosis and vascular dysfunction. Second, and perhaps most importantly, we provide direct evidence linking *IL-1β* to CMD. The significant negative correlation between *IL-1β* expression and CFR suggests that the inflammatory milieu driven by *IL-1β* impairs the function of the coronary microvasculature, leading to myocardial ischemia and contributing to the heightened risk of MACE.

We acknowledge several limitations in our study. First, as a single-center, observational study, our findings may have limited generalizability and cannot establish causality. The associations observed require validation in larger, multi-center cohorts. Second, although we adjusted for numerous potential confounders, residual confounding cannot be entirely excluded. Third, the gene expression was measured at a single time point, which may not fully capture the dynamic nature of the inflammatory state over the long-term follow-up period. Finally, while our data strongly suggest a link between *IL-1β* and CMD, we did not directly assess the impact of *IL-1β*-targeted therapies on coronary microvascular function.

In conclusion, this study establishes monocytic *IL-1β* expression as a robust, independent predictor of MACE and mortality in KTRs, an association that may be mediated by more severe CMD among patients with higher *IL-1β* levels.

## Data Availability

The datasets presented in this article are not readily available because of patient privacy and confidentiality concerns. Requests to access the datasets should be directed to Chang'an Wang, drwangchangan@163.com.

## Supplementary material

Supplementary Figure S1 Non-linear association of *IL-1β* expression with death and MACE. Restricted cubic spline plots showing the hazard ratio and 95% confidence intervals (CI) for **(A)** all-cause death and **(B)** major adverse cardiovascular events (MACE) according to the continuous relative expression of *IL-1β*. The red dashed line represents a hazard ratio of 1.0 (no effect). *P*-values for the overall association and for non-linearity are provided.
