# Supplementary material for: Monocyte IL-1β predicts adverse cardiovascular events and associates with coronary microvascular dysfunction in kidney transplant recipients
Source: Front Cardiovasc Med. 2026 Jan 7;12:1689566. doi: 10.3389/fcvm.2025.1689566 (PMC12819680; doi:10.3389/fcvm.2025.1689566)
Supplement: Supplementary file 1 [file Table1.docx]

**Table S1. Endpoints Definition**

| **Endpoints** | **Definition** |
| --- | --- |
| **All-cause Death** | Death of any cause. |
| **Nonfatal MI** | Meeting all the 3 following criteria: |
|  | 1. Detection of a rise and/or fall of cTn values with at least one value above the 99th percentile URL; |
|  | 2. Meeting at least one of the following criteria: |
|  | - Symptoms of myocardial ischemia; |
|  | - New ischemic ECG changes; |
|  | - Development of pathological Q waves; |
|  | - Imaging evidence of new loss of viable myocardium or new regional wall motion abnormality   in a pattern consistent with an ischemic aetiology; |
|  | - Identification of a coronary thrombus by angiography; |
|  | - Stent thrombosis or restenosis. |
|  | 3. Excluding death, PCI related MI, and CABG related MI. |
| **Nonfatal Stroke** | Meeting all the 3 following criteria: |
|  | 1. Defined as the presence of a new focal neurologic deficit thought to be vascular in origin, with signs or symptoms lasting more than 24 hours; |
|  | 1. Radiological evidence from CT or MRI scans; 2. Excluding death. |
| **Hospitalization due to HF** | Clinically diagnosed acute heart failure requiring hospitalization for >72 hours and treatment with diuretics by intravenous injection. |

CABG, coronary artery bypass grafting; CT, computed tomography; ECG, electrocardiogram; HF, heart failure; MI, myocardial infarction; MRI, magnetic resonance imaging; PCI, percutaneous coronary intervention; URL, upper reference limitation.
