# Supplementary figures and images for: Monocyte IL-1β predicts adverse cardiovascular events and associates with coronary microvascular dysfunction in kidney transplant recipients
Source: Front Cardiovasc Med. 2026 Jan 7;12:1689566. doi: 10.3389/fcvm.2025.1689566 (PMC12819680; doi:10.3389/fcvm.2025.1689566)

**A****Death**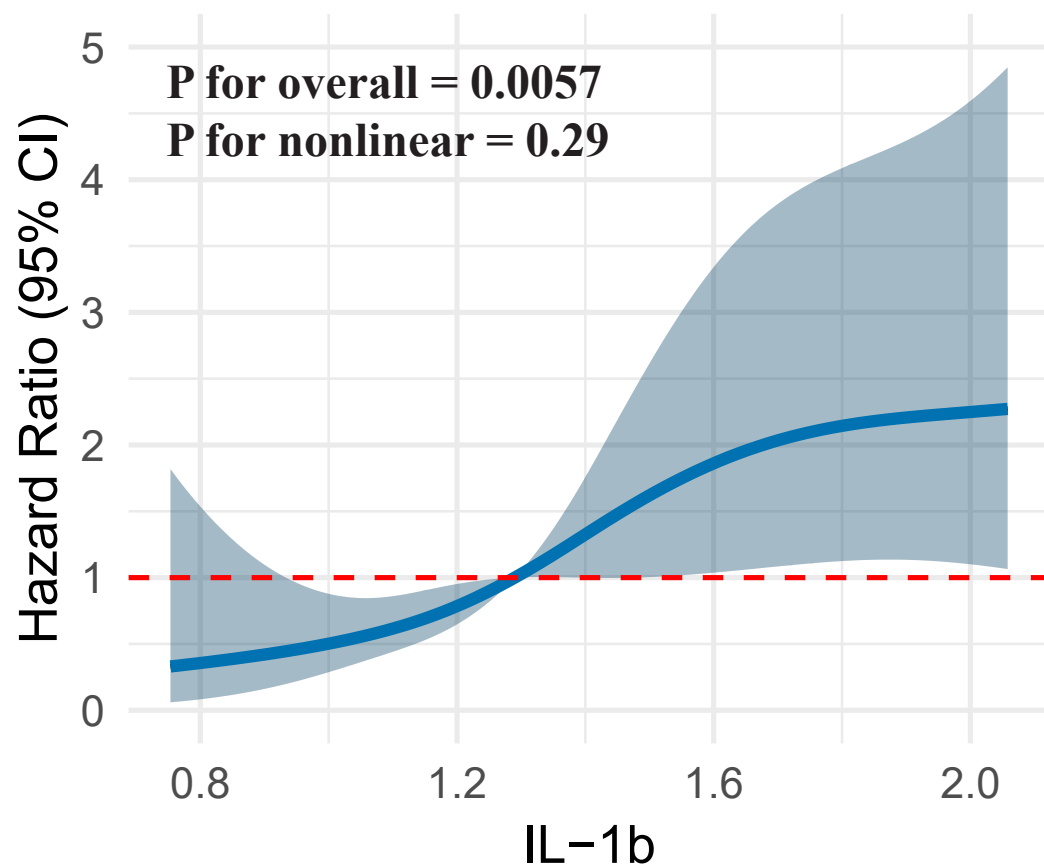**B****MACE**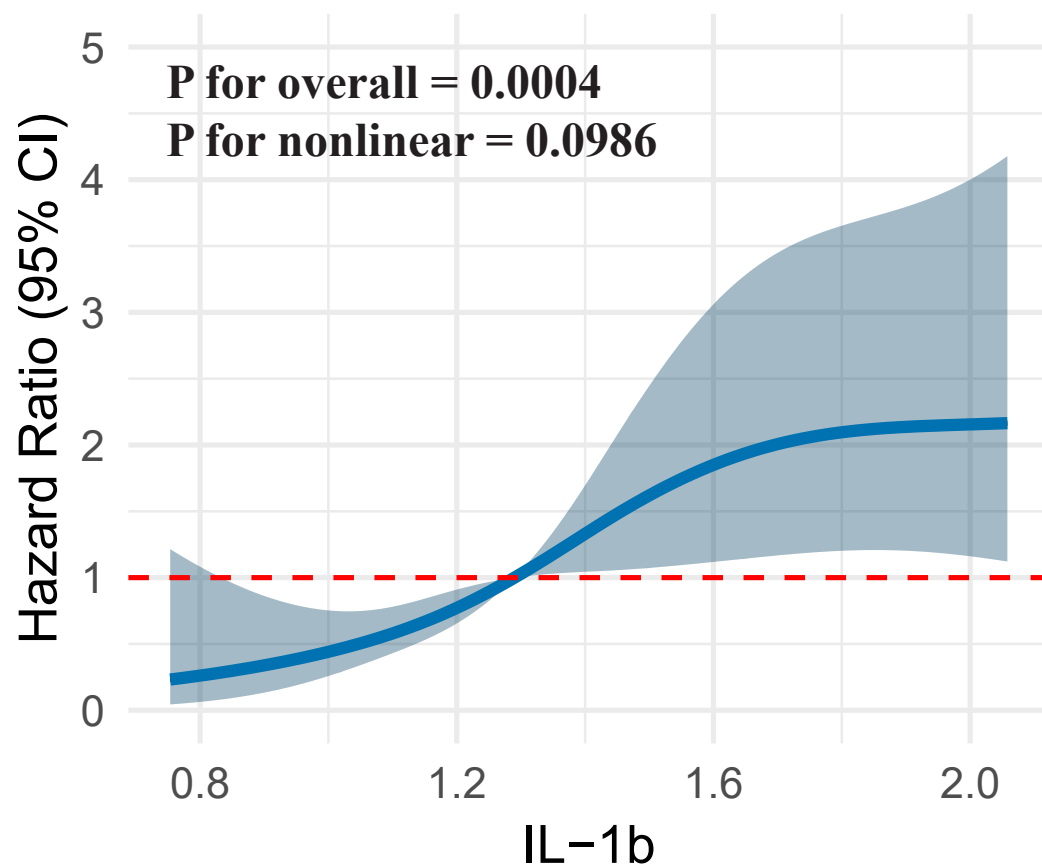

Supplement: Supplementary Figure S1 — Non-linear association of IL-1β expression with death and MACE. Restricted cubic spline plots showing the hazard ratio and 95% confidence intervals (CI) for (A) all-cause death and (B) major adverse cardiovascular events (MACE) according to the continuous relative expression of IL-1β. The red dashed line represents a hazard ratio of 1.0 (no effect). P-values for the overall association and for non-linearity are provided. [file Image1.pdf]
